# The Oncology Association of Bosnia and Herzegovina’s recommendations for fertility preservation in oncologic patients

**DOI:** 10.17305/bjbms.2021.6977

**Published:** 2022-03-15

**Authors:** Timur Cerić, Emir Sokolović, Berisa Hasanbegović, Anes Pašić, Zdenka Gojković, Jelena Vladičić Mašić, Nikolina Dukić, Inga Marijanović, Alma Mekić Abazović, Ibrahim Šišić, Dijana Koprić, Mustafa Hammami, Senad Bajramović, Taib Delić, Semir Bešlija

**Affiliations:** 1 Clinic of Oncology, Clinical Center University of Sarajevo, Sarajevo, Bosnia and Herzegovina; 2 Oncology Clinic, Clinical Center of Republic of Srpska, Banja Luka, Bosnia and Herzegovina; 3 Department of Oncology, University Hospital Foča, Foča, Bosnia and Herzegovina; 4 Oncology Clinic, University Clinical Hospital Mostar, Mostar, Bosnia and Herzegovina; 5 Department of Oncology, Cantonal Hospital Zenica, Zenica, Bosnia and Herzegovina; 6 Department of Oncology, University Clinical Center Tuzla, Tuzla, Bosnia and Herzegovina; 7 Department of Oncology, General hospital “Dr. Irfan Ljubijankić” Bihać, Bihać, Bosnia and Herzegovina; 8 Clinic of Urology, Clinical Center University of Sarajevo, Sarajevo, Bosnia and Herzegovina; 9 Polyclinic Sunce, Sarajevo, Bosnia and Herzegovina

**Keywords:** Cryopreservation, fertility preservation, neoplasms, oncofertility, oncology

## Abstract

Malignancy is one of the major public health problems in Bosnia and Herzegovina. Along with breakthroughs in specific oncological therapy, improving the quality of life of cancer patients and management of therapy-induced side effects need to be recognized as a priority in the comprehensive cancer patient care. Fertility loss after cancer treatment is a field requiring special attention due to its various consequences on patients themselves. Although oncofertility is well-recognized area of oncology, low- to middle-income countries are facing issues with its implementation in everyday practice. Increased awareness about fertility preservation is of high priority for all specialists who participate in the medical care of cancer patients. The absence of a systemic solution and lack of expertise led to the founding of Fertility Preservation Working Group of the Oncology Association of Bosnia and Herzegovina. We have made recommendations as an expert consensus with the ultimate goal of making the first step toward enhancement of oncofertility implementation in Bosnia and Herzegovina.

## INTRODUCTION

Malignancy is one of the major public health problems in Bosnia and Herzegovina. Based on the data presented in the Globocan report, there were 14,673 new cases of cancer during 2020. The most common cancer types by site were lung cancer (17.1%), colorectal cancer (12.7%), breast cancer (10.6%), prostate cancer (6.1%), and gastric cancer (5.2%) [1].

Improved treatment of malignant diseases in recent decades has resulted in better survival of patients and more successful management of symptoms caused by the disease itself. However, it should be recognized that improving the quality of life of cancer patients and controlling therapeutic side effects are also priorities in cancer therapy.

Sterility after cancer therapy is one of the most significantly reported problems and requires special attention given the biological and psychological consequences whose severity is immeasurable [2]. Oncofertility is an emerging field of medicine whose main goal is to improve the quality of life of oncology patients by increasing access to opportunities to preserve fertility [3].

Although oncofertility plays an important role in oncology therapy in developed countries, the implementation of oncofertility is still problematic in moderate- and low-income countries, especially in terms of resources, expertise, and costs [4]. In a study of data from 40 centers around the world, participants responded that the most common barriers to implementing oncofertility were financial burdens for patients (62%), religious or cultural constraints (61%), and a lack of specialized providers/health facilities (24%) [5].

The Fertility Preservation Working Group of the Oncology Association of Bosnia and Herzegovina was established and launched its activities for several reasons; primarily due to the increase in the number of cancer patients of a reproductive age, but also due to the need to establish diagnostic and therapeutic recommendations, to facilitate cooperation and work for patients and healthcare professionals (oncologists, gynecologists, urologists, etc.) increased the quality of care for our patients. It is important to know if the patient should be referred to a specialist or a reproductive center. There are very few centers in Bosnia and Herzegovina that deal with fertility procedures, and these centers are mostly private health institutions- meaning that patients are forced to bear the financial costs of such procedures due to lack of health insurance coverage. Aforementioned factors contribute to the psychological and financial stress associated with the treatment of malignant disease, i.e., the decline in the quality of life of patients. An additional goal of the guidelines is to inform decision-makers related to this issue and to find a systemic solution that diminishes stress (psychological and financial) that accompanies malignant disease (Table 1).

**TABLE 1:**
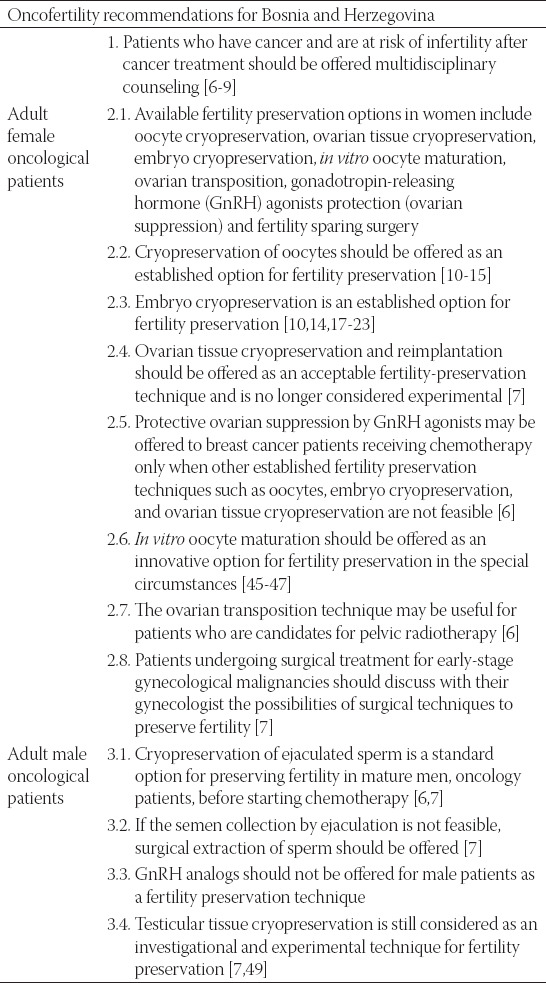
Oncofertility recommendations for Bosnia and Herzegovina

## ONCOFERTILITY RECOMMENDATIONS FOR BOSNIA AND HERZEGOVINA

### Recommendation 1

Patients who have cancer and are at risk of infertility after cancer treatment should be offered multidisciplinary counseling. Discussion should be started as soon as possible, before initiating treatment and this should be documented in the patient’s record [6-9].

## PRESERVATION OF FERTILITY IN ADULT FEMALE ONCOLOGICAL PATIENTS – RECOMMENDATIONS

### Recommendation 2.1

Available fertility preservation options in women include oocyte cryopreservation, ovarian tissue cryopreservation (OTC), embryo cryopreservation, *in vitro* oocyte maturation (IVM), ovarian transposition, gonadotropin-releasing hormone (GnRH) agonists protection (ovarian suppression), and fertility sparing surgery.

### Recommendation 2.2

Cryopreservation of oocytes should be offered as an established option for fertility preservation [10-15]. Cryopreservation of oocytes, along with cryopreservation of embryos, is considered the “gold standard” for preserving fertility. The oocyte cryopreservation technique requires about two weeks to stimulate the oocytes before storing them, so the start of specific cancer treatment could be delayed [8]. The live birth rate as a measure of oocyte cryopreservation success published in Cobo *et al*. (2016) is 50% in women not older than 35 years and 22.9% in women older than 36 years [16].

### Recommendation 2.3

Embryo cryopreservation should be offered as an established fertility preservation option [10,14,17-23]. Ovarian stimulation is required, and this procedure takes an average of two weeks [8]. The live birth rate as a measure of embryo cryopreservation success ranges from 20% to 45% [24,25].

### Recommendation 2.4

OTC and reimplantation should be offered as an acceptable fertility-preservation technique and it is no longer considered experimental [7]. This technique is of the utmost importance for patients who need to start cancer treatment urgently, because ovarian stimulation is not needed, as well as for prepubertal patients for whom OTC is the only option for preserving fertility [8]. Reported live birth rates in the OTC literature from selected studies range from 18.2% to 40% [24,26-30]. Ovarian reimplantation is considered safe in terms of the risk of reintroducing cancer cells into the body, provided that previous pelvic invasion is ruled out [31]. After reimplantation of ovarian tissue, a five-year oncological monitoring is recommended [32]. Due to the higher risk of ovarian cancer in patients with the BRCA mutation, OTC for transplantation is not recommended [7].

### Recommendation 2.5

Protective ovarian suppression by GnRH agonists may be offered to breast cancer patients receiving chemotherapy only when other established fertility preservation techniques such as oocytes, embryo cryopreservation, and OTC are not feasible [6]. Several randomized studies and meta-analyses have evaluated the efficacy of GnRH agonists for ovarian protection, but the results have been inconsistent [33-44].

### Recommendation 2.6

IVM should be offered as an innovative option to preserve fertility in exceptional circumstances [45-47]. It should be offered to patients, in whom there is not enough time for ovarian stimulation, but in whom there is an indication for cryopreservation of oocytes. This technique should be discussed with patients who carry BRCA mutations at the time of oophorectomy if other fertility options are not feasible [7].

### Recommendation 2.7

The ovarian transposition technique may be useful for patients who are candidates for pelvic radiotherapy. Patients should be informed of the limited success of this technique due to unpredictable damage of radiation dissipation [6].

### Recommendation 2.8

Patients undergoing surgical treatment of early gynecological malignancies should discuss with their gynecologist the possibilities of surgical techniques to preserve fertility [7].

## PRESERVATION OF FERTILITY IN ADULT MALE ONCOLOGICAL PATIENTS – RECOMMENDATIONS

### Recommendation 3.1

Cryopreservation of ejaculated sperm is a standard option for preserving fertility in mature male oncology patients, before starting chemotherapy [6,7]. If collecting semen by masturbation is not feasible, patients should be advised to discuss with their urologist other available options such as the use of phosphodiesterase type 5 inhibitors, vibration stimulation, electroejaculation, or alpha-agonist therapy for patients with retrograde ejaculation^.^ [7].

### Recommendation 3.2

If collecting semen through ejaculation is not feasible, patients should be offered surgical sperm extraction. This technique is extremely important for patients with single testicles or contralateral testicular atrophy, because this group of patients is at higher risk of azoospermia [7]. Testicular sperm extraction with an operating microscope can be particularly useful for identifying focal areas with active spermatogenesis, as this technique can ensure greater efficacy in oncology patients [48].

### Recommendation 3.3

GnRH analogs should not be offered for male patients as a fertility preservation technique, because human studies have not shown the effectiveness of this procedure.

### Recommendation 3.4

Cryopreservation of testicular tissue is still considered an experimental technique for preserving fertility. It is the only option for preserving fertility for patients in the prepubertal period and should be offered only through clinical trials [7,49].

## CONCLUSION

Increasing awareness of fertility preservation and willingness to discuss this topic is a priority for all specialists involved in the medical care of cancer patients. These recommendations were given by the Working Group for Preservation of Fertility of the Oncology Association in Bosnia and Herzegovina as an expert consensus with the goal to be the first step in improving the implementation of oncofertility in Bosnia and Herzegovina. We strongly encourage the launch of clinical trials and research projects aimed at clarifying and defining fertility preservation options for cancer patients in our country.
